# Digital Manufacturing of Selective Porous Barriers in Microchannels Using Multi-Material Stereolithography

**DOI:** 10.3390/mi9030125

**Published:** 2018-03-14

**Authors:** Yong Tae Kim, Kurt Castro, Nirveek Bhattacharjee, Albert Folch

**Affiliations:** Department of Bioengineering, University of Washington, Seattle, WA 98195, USA; castrokb@uw.edu (K.C.); nirveek@uw.edu (N.B.); afolch@uw.edu (A.F.)

**Keywords:** multi-material stereolithography, porous barrier, diffusion, microfluidics

## Abstract

We have developed a sequential stereolithographic co-printing process using two different resins for fabricating porous barriers in microfluidic devices. We 3D-printed microfluidic channels with a resin made of poly(ethylene glycol) diacrylate (MW = 258) (PEG-DA-258), a UV photoinitiator, and a UV sensitizer. The porous barriers were created within the microchannels in a different resin made of either PEG-DA (MW = 575) (PEG-DA-575) or 40% (*w*/*w* in water) PEG-DA (MW = 700) (40% PEG-DA-700). We showed selective hydrogen ion diffusion across a 3D-printed PEG-DA-575 porous barrier in a cross-channel diffusion chip by observing color changes in phenol red, a pH indicator. We also demonstrated the diffusion of fluorescein across a 3D-printed 40% PEG-DA-700 porous barrier in a symmetric-channel diffusion chip by measuring fluorescence intensity changes across the porous barrier. Creating microfluidic chips with integrated porous barriers using a semi-automated 3D printing process shortens the design and processing time, avoids assembly and bonding complications, and reduces manufacturing costs compared to micromolding processes. We believe that our digital manufacturing method for fabricating selective porous barriers provides an inexpensive, simple, convenient and reproducible route to molecule delivery in the fields of molecular filtration and cell-based microdevices.

## 1. Introduction

Precise control of small molecule diffusion through porous materials and its application to analyze cell behavior is an important technology in fundamental biology, biomedicine, and pharmaceutics [1,2,3,4,5]. Lab-on-a-chip research has progressed over the last decades by adapting microfabrication technology from semiconductor fabrication processes that bring advantages such as miniaturization, uniformity, accuracy, reproducibility, and fluid/cell/tissue manipulations [6,7,8,9,10,11,12]. Hydrogels, which are three-dimensional (3D) polymer networks with high porosity, have been employed in the field of tissue engineering, drug delivery systems, biomaterials, and size-selective separation, among others [13,14,15,16,17,18]. The pore size of hydrogels can be easily tuned by manipulating their crosslinking density.

Researchers have used microfluidic architectures in combination with hydrogel materials for delivery of chemicals in cell and tissue engineering applications [19,20,21,22]. For example, Lee et al. developed a macroporous polyacrylamide and poly(ethylene glycol) diacrylate (PEG-DA) hydrogel array within a microchannel to monitor solute transport into the hydrogel; they fabricated macroporous hydrogels composed of polyacrylamide and analyzed the diffusion of both glutathione *S*-transferase-green fluorescent protein and dextran-fluorescein isothiocyanate into the hydrogel; they also produced PEG-DA (MW = 700) (PEG-DA-700) hydrogels and tested the penetration of 250 kDa dextran into the hydrogel [23]. Huang et al. reported a 3D microfluidic platform, which was fabricated using typical photolithography methods, to observe cell to extracellular matrix and cell to cell interactions; they filled multiple adjacent channels with type-I collagen and Matrigel by balancing the capillary forces and surface tension; the different patterned gels in the microchannels enabled real-time observation of intercellular interactions in co-cultures using MDA-MB-231 cell and RAW cells [24]. Sung et al. announced a microfluidic device for testing drug toxicity in a pharmacokinetic-based manner that included multiple hydrogel-based cell culture chambers connecting to microfluidic channels to mimic multi-organ interactions [25]. However, fabrication of porous microstructures in a microfluidic chip usually requires the assembly and bonding of an additional membrane or hydrogel structure, a process that is complex, costly, and time-consuming, and often requires the intervention of a trained specialist.

3D printing has emerged as an attractive alternative technique for manufacturing microfluidic devices because of its attributes, i.e., 3D design, semi-automated and assembly-free 3D fabrication, rapidly decreasing costs, and faster production time [26,27]. 3D printing has also been extensively used to build hydrogel scaffolds [28,29]. For example, Mohanty et al. fabricated scalable scaffolds using a combined 3D-fused deposition modeling printing and salt leaching process for precise control of the geometry and dimensions of the scaffolds [30,31]. He et al. investigated the printability of a sodium alginate and gelatin mixture hydrogel using an extrusion-based 3D bioprinting system and demonstrated the viability of L929 mouse fibroblasts on the 3D-printed hydrogel scaffold [32]. Hinton et al. recently reported a complex 3D biological structure using a thermoreversible gelatin support bath that enabled freeform reversible embedding of hydrogel to suspend alginate, fibrin, collagen type I, and Matrigel [33]. However, multi-material printing has not been used yet for the production of porous barriers in microfluidics. 

Integrating the fabrication of a porous microstructure within a microfluidic device using an automated process such as 3D printing allows for materials and performance modeling prior to fabrication, shortens the duration of design iterations, and reduces overall manufacturing costs. In this work, we developed a stereolithographic co-printing process to enable the inexpensive microfabrication of selective porous barriers in order to control the diffusion of small ions or molecules within a microchannel. We explored two different hydrogels—PEG-DA (MW = 575) (PEG-DA-575) and 40% (*w*/*w* in water) PEG-DA-700 (40% PEG-DA-700)—for printing the porous barriers separating two adjacent microchannels. The microchannels were fabricated with PEG-DA (MW = 258) (PEG-DA-258) [34]. Diffusion of hydrogen ions and fluorescein was demonstrated with a 3D-printed cross-channel diffusion chip (PEG-DA-575 barrier) and a 3D-printed symmetric-channel diffusion chip (40% PEG-DA-700 barrier), respectively.

## 2. Materials and Methods

### 2.1. Resin Composition for Multi-Material Stereolithography Printing

Photocurable resins for stereolithography printing were developed for the microfluidic chips that have selective porous barriers within their microchannels. The resin for creating the microchannel floor, walls, and roof is based on PEG-DA-258 (Sigma Aldrich, St. Louis, MO, USA) mixed with 0.6% (*w*/*w*) Irgacure 819 (IRG, BASF Corporation, Florham Park, NJ, USA) as a photoinitiator and 0.6% (*w*/*w*) 2-isopropylthioxanthone (ITX) as a photosensitizer. The resin for the porous barrier of the 3D-printed cross-channel diffusion chip contains PEG-DA-575 (Sigma Aldrich), 0.6% IRG, and 0.6% ITX. 40% PEG-DA-700 (Sigma Aldrich) in distilled water conjugated with 0.6% IRG is used for printing the porous barrier of the 3D-printed symmetric-channel diffusion chip.

### 2.2. Stereolithography—Setup and Printing

Both the cross-channel and symmetric-channel diffusion chip were designed with Autodesk Inventor^®^ and saved in STL formats. In order to slice the designed object, we used Autodesk Print Studio (Autodesk, Inc., San Rafael, CA, USA), which has a function for automatic mesh analysis and healing. After slicing the designed object into 25 μm-thick layers, the sliced images and the UV exposure settings for each layer outlined in a CSV file named layersettings.csv were compressed into a zipped folder. The folder was then sent to the 3D printer to fabricate the object. The fabrication of the multi-material diffusion microfluidic chip was carried out in bat configuration with an Ember DLP^®^ 3D Printer (Autodesk, Inc.), which has a 405 nm wavelength LED, 50 μm in-plane resolution, and 10~100 μm out-of-plane resolution controlled by a digital light projector (DLP). To make transparent prints, we used a glass slide (75 mm (L) × 50 mm (W) × 1.0 mm (T)) as the substrate on which the designed object was built [34]. Prior to using the glass slide, the glass surface was thoroughly cleaned with acetone, isopropyl alcohol, and deionized (D.I.) water and dried at 70 °C overnight. The surface of the glass slide was plasma-activated with oxygen plasma for 180 s and treated with 3-(trimethoxysilyl) propyl methacrylate (TMSPMA) (Sigma-Aldrich) in an 85 °C chamber for 8 h. The silanized glass slide was attached to the aluminum build plate by coating one side of it with uncured PEG-DA-258 resin and exposing it to UV light using a broadband UV lamp (B-100 A, UVP). For the microchannel fabrication, the PEG-DA-258 resin was poured into a resin tray and then the build plate was lowered until it reached the resin tray for calibration. After calibration, the object was printed with repeated cycles of UV curing, resin tray separation from the current print position, Z-Axis overlift, and resin tray approach to the new print position. This process was repeated until the whole object was fabricated. For multi-material printing, the printer was paused at the desired layer and the PEG-DA-258 resin tray was changed to the other material (PEG-DA-575 or 40% PEG-DA-700), or vice versa. The glass slide with the printed object was removed from the build plate and cleaned using D.I. water and pressurized air.

### 2.3. Molecule Diffusion Analysis

To test the hydrogen ion diffusion through the porous barrier printed using PEG-DA-575 in the 3D-printed cross-channel diffusion chip, phenol red (Sigma) was employed to observe color changes corresponding to changes in acidity. Then 0.1 M HCl was injected into the bottom channel of the cross-channel diffusion chip and phenol red was loaded into the top channel of the diffusion chip. The color change of the phenol red was recorded using a Nikon SMZ1500 stereomicroscope (Nikon, Tokyo, Japan) fitted with a Canon Rebel EOS 5D Mark II DSLR camera (Canon, Tokyo, Japan).

The 3D-printed symmetric-channel diffusion microchip was utilized to transport fluorescein molecules through its porous barrier made of 40% PEG-DA-700 resin. The 0.1 M fluorescein was inserted into Channel 1 of the symmetric-channel diffusion chip while PBS buffer was loaded into Channel 2. The diffusion of fluorescein across the porous barrier from one channel to the other was measured for 1 hour using an inverted epifluorescence microscope (Nikon Eclipse Ti, Plan Apo λ 2× objective).

## 3. Results and Discussion

### 3.1. Selection of Resin Components

We have fabricated selective porous barriers in microchannels using multi-material stereolithography. We chose PEG-DA-258 to print microfluidic channels due to its characteristics such as photo-curability, water impermeability, transparency, and low cost [34]. We added 0.6% IRG to the PEG-DA-258 resin because of its ability to absorb energy and initiate free radical polymerization at longer UV wavelengths (405 nm), corresponding to that of the printer DLP projector. We also added ITX at 0.6% to increase the Z-resolution of 3D-printed microchannels. ITX, which is a sulfur-type photosensitizer, is widely used in UV curing to promote the polymerization of photopolymers because of its UV absorption characteristics. PEG-DA with higher molecular weights were chosen as porous barrier materials since it had been shown before that PEG-DA hydrogel networks (MW = 575 to 20,000) have a mesh size of 0.1 to 10 nm [35,36,37]. A porous barrier in the cross-channel diffusion chip was printed using PEG-DA-575 mixed with 0.6% IRG and 0.6% ITX for small ion diffusion such as hydrogen ions. We also used 40% PEG-DA-700 in D.I. water containing 0.6% IRG to make a porous barrier for the transport of larger molecules since adding 60% water to PEG-DA-700 resin increases the pore size in a hydrogel polymer matrix [16].

### 3.2. Fabrication of a 3D-Printed Cross-Channel Diffusion Chip

**Figure 1:**
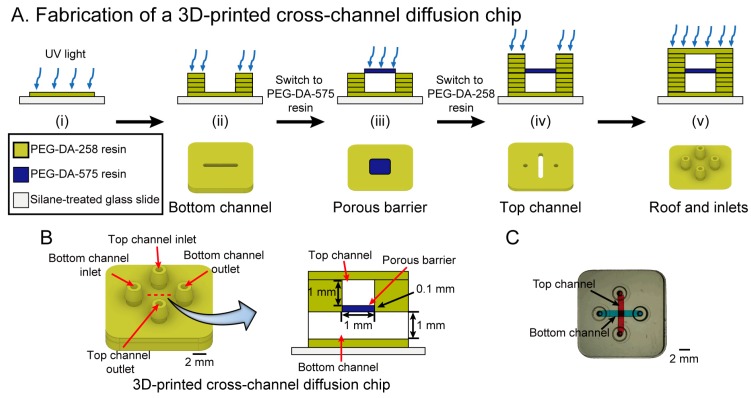
(**A**) Fabrication process in cross-section schematics (top row) and 3D view (bottom row) of a 3D-printed cross-channel diffusion microchip containing a PEG-DA-575 porous barrier. The process depicts the fabrication of (i) the bottom layer; (ii) the bottom channel; (iii) the porous barrier; (iv) the top channel; and (v) the roof and inlets; (**B**) CAD representation of the finished 3D-printed cross-channel diffusion chip (left) and its cross-sectional schematic (right); (**C**) photograph (top view) of the 3D-printed cross-channel diffusion chip; the top channel is filled with red dye and the bottom channel is filled with blue dye for visualization purposes.

Figure 1A shows the fabrication process of a cross-channel diffusion chip. We developed two different resins to print the 3D-printed cross-channel diffusion chip: a resin to build the porous barrier (PEG-DA-575) and a microfluidic channel resin (PEG-DA-258). Prior to the printing of the 3D-printed cross-channel diffusion chip, the designed object was sliced into 25 μm-thick layers. A PEG-DA-258 resin tray and a PEG-DA-575 resin tray were prepared to build the microchannel part and the porous barrier part, respectively. First, the PEG-DA-258 resin tray was inserted into the Ember DLP^®^ 3D Printer and the first layer was created with 6 s UV exposure to aid the adhesion of the device to the glass slide. After printing a 100 μm-thick bottom layer, a 1 mm high bottom channel was fabricated using the PEG-DA-258 resin with a 0.3 s exposure setting for each layer. Prior to build the porous barrier, PEG-DA-258 residue was washed using D.I. water. The PEG-DA-575 resin tray was inserted into the printer for fabricating the porous barrier. The 100 μm-thick porous barrier was printed by irradiating each layer for 0.8 s. Compared to PEG-DA-258, PEG-DA-575 needs longer exposure times to fabricate polymer structures because, for the same polymer mass, PEG-DA-575 has less active sites (diacrylate groups) for photopolymerization than PEG-DA-258; the smaller number of active sites results in a slower reaction speed in PEG-DA-575. The lower molecular weight monomers in PEG-DA-258 have a higher mobility in the resin, which increases the probability of radical initiation on the carbon-carbon double bond (this radical initiation is the first step of the polymerization process). Moreover, due to its higher mobility compared to PEG-DA-575, the PEG-DA-258 molecule has a greater chance of encountering other molecules, which also results in a faster reaction speed. To fabricate a 1 mm-high top channel, the resin tray was switched back to the PEG-DA-258 resin tray after rinsing the PEG-DA-575 residue and the top channel was fabricated with the same conditions as the bottom channel. In order to prevent the microchannel from clogging with PEG-DA-258 residue due to overexposure, the resin in the microchannels was removed after printing a 100 μm-thick top channel roof and the fabrication continued until the diffusion chip was completed. At the end of the print process, the microchannels were flushed in a dark room with distilled water to remove any PEG-DA resin from the channel.

Figure 1B illustrates a CAD design of the 3D-printed cross-channel diffusion chip and its cross-sectional view. A photo image of the 3D-printed cross-channel diffusion chip is displayed in Figure 1C. The bottom channel is filled with blue dye while the top channel is filled with red dye.

### 3.3. Hydrogen Ion Diffusion Test

**Figure 2:**
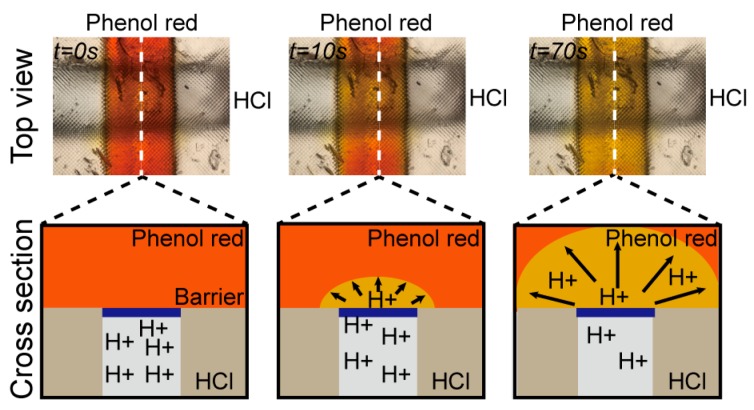
Hydrogen ion diffusion experiment (**top row**) and cross-section schematic (**bottom row**) using the 3D-printed cross-channel diffusion chip. The phenol red in the channel (*t* = 0 s) gradually changed to yellow at the channel intersection (*t* = 10 s) until it turned completely yellow (*t* = 70 s), while the bottom channel did not change color.

We confirmed the selective diffusion of hydrogen ions through a PEG-DA-575 barrier by observing pH shifts as color changes with the pH-sensitive phenol red dye. The phenol red color was shown to gradually shift from yellow to red over the pH range 6.8 to 8.2 [38]. 0.1 M HCl solution was injected into the bottom channel of the cross-channel diffusion chip and then 0.5% phenol red in PBS was loaded into the top channel. Hydrogen ion diffusion was recognized by observing the color change of phenol red as shown in Figure 2. The phenol red solution at 0 s gradually took on a yellow color at 10 s at the porous barrier area, corresponding to the overlap between the two microchannels. The phenol red turned completely yellow after 70 s. The color change happened only in the top channel, which means hydrogen ions can pass through the porous barrier from the bottom channel to the top channel, while phenol red remains in the top channel, as illustrated in the cross-section view of Figure 2.

### 3.4. Fabrication of a 3D-Printed Symmetric-Channel Diffusion Chip

Small-ion diffusion was successfully carried out with the 3D-printed cross-channel diffusion chip (see Section 3.3). However, some biomedical applications may require the delivery of larger molecules such as dyes and drugs. To make a hydrogel barrier with larger pores, we used 40% PEG-DA-700 mixed with 60% water. The higher molecular weight of PEG-DA can produce larger pores in the hydrogel because of its longer chain length, which increases the mesh size of the polymer network. To polymerize a dilute PEG-DA-700 resin, a longer exposure time is required, which increases the risk of clogging channels under the porous barrier. We changed the diffusion chip design to a symmetric-channel chip having a porous barrier in between two channels, which ensured that the longer exposure time required to polymerize 40% PEG-DA-700 did not occlude the channels.

**Figure 3:**
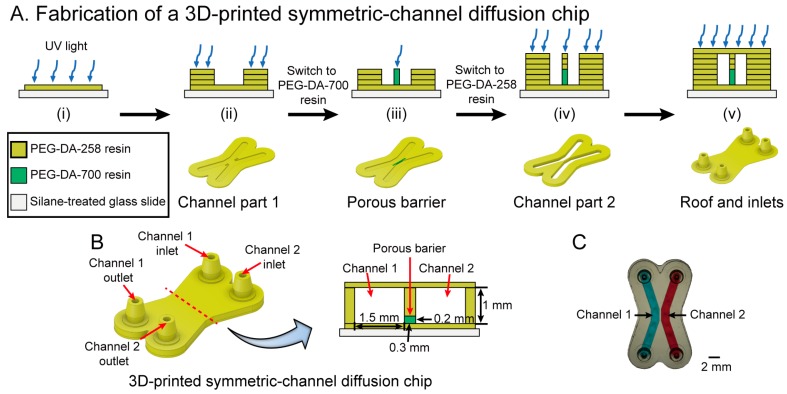
(**A**) Fabrication process in cross-section schematics (**top row**) and 3D view (**bottom row**) of a 3D-printed symmetric-channel diffusion microchip with a 40% *w*/*w* PEG-DA-700 porous barrier. The process depicts the fabrication of (i) the bottom layer; (ii) the channel part 1; (iii) the porous barrier; (iv) the channel part 2; and (v) the roof and inlets; (**B**) CAD representation of the 3D-printed symmetric-channel diffusion chip (**left**) and its cross-sectional schematic (**right**); (**C**) photograph of the 3D-printed symmetric-channel diffusion chip; Channel 1 is filled with blue dye and Channel 2 is filled with red dye for visualization purposes.

The fabrication process of a 3D-printed symmetric-channel diffusion chip is described in Figure 3A. PEG-DA-258 resin and 40% PEG-DA-700 resin in D.I. water were used to print the symmetric-channel and porous barrier, respectively. Prior to printing, the 3D designed symmetric-channel diffusion chip was sliced into 25 μm thick layers similar to the cross-channel chip. As explained before, a 100 μm-thick bottom layer was printed with 6 s exposure and then eight layers of the channel (200 μm) were fabricated with 0.3 s UV irradiation for each layer. Next, the PEG-DA-258 residue was washed using D.I. water and the 40% PEG-DA-700 resin tray was inserted. The porous barrier was created by a single 6.5 s exposure of the resin, which allowed the PEG-DA-700 hydrogel structure to grow down and attach to the glass slide. The porous barrier is designed as the shape of an extruded rectangle between two microchannels and is, by design, 200 μm tall, 300 μm wide and 2 mm long. The PEG-DA-258 resin tray was re-installed in the 3D printer to build the last of the channel layers after cleaning the PEG-DA-700 residue. To avoid clogging of the microchannels, they were cleaned after printing a 100 μm-thick roof layer and then the diffusion chip fabrication was completed. The microchannels were washed using distilled water to remove uncured PEG-DA resin. Figure 3B,C show a schematic cross section and a photograph, respectively, of the device.

### 3.5. Fluorescein Diffusion Test

**Figure 4:**
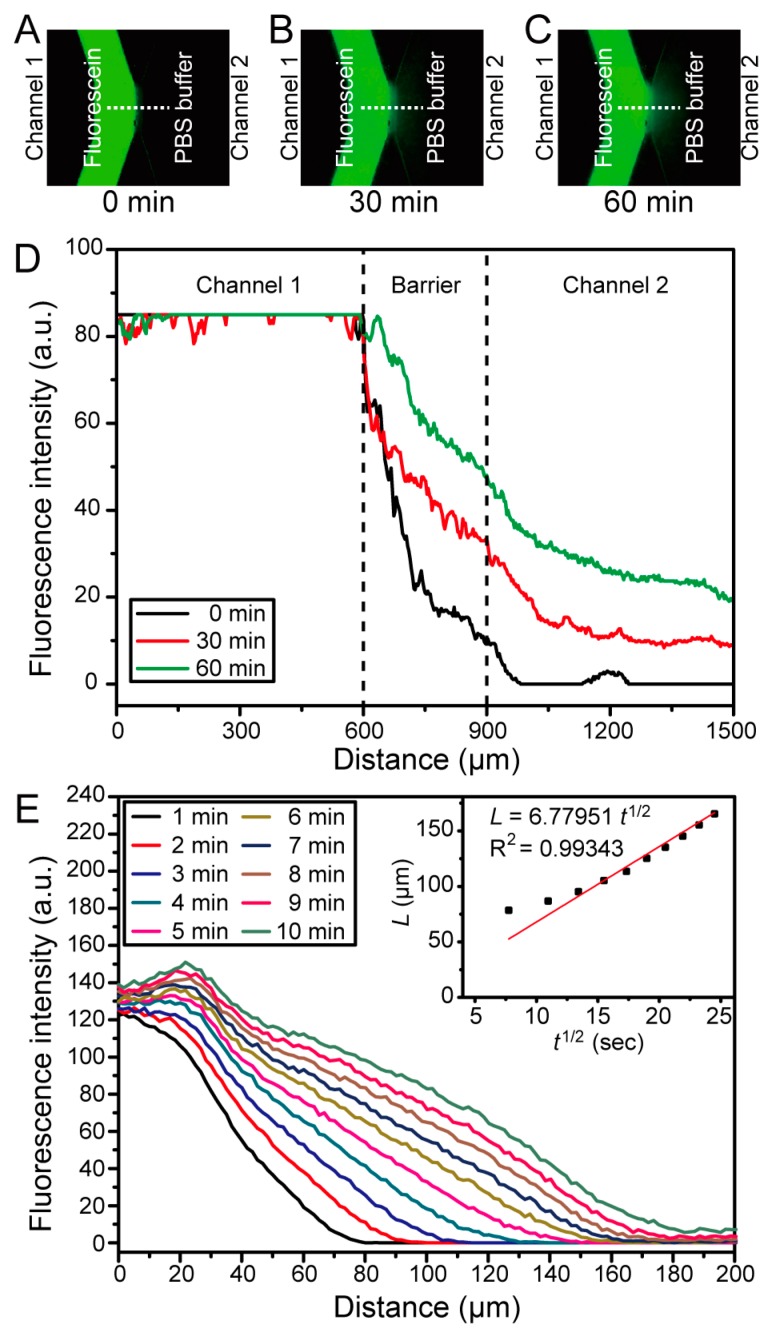
Fluorescein diffusion test using the 3D-printed symmetric-channel diffusion microchip. (**A**–**C**) Fluorescein diffused through the 40% PEG-DA-700 hydrogel barrier from 0 min to 60 min; (**D**) graph of the fluorescein intensity profiles obtained across the microchannel in the three images above, showing that the fluorescein intensity gradually increased in Channel 2; (**E**) concentration profile of fluorescein in the 40% PEG-DA-700 hydrogel barrier from 1 min to 10 min. (Inset) Plot of the characteristic diffusion length as a function of the square root of time (dots); the red line is a linear fit of the dots, L = 6.77951t12 (adjusted *R*^2^ = 0.99343).

Molecular diffusion across the 40% PEG-DA-700 barrier in the 3D-printed symmetric-channel chip was tested by observing the transport of fluorescein (as a model of a small drug) using an inverted epifluorescence microscope. 0.1 mM fluorescein was loaded into Channel 1 while PBS buffer was loaded into Channel 2. The change in fluorescence intensity as fluorescein diffused from Channel 1 to Channel 2 was measured for 1 h. The results of the fluorescein diffusion are shown in Figure 4. The green signal was observed only in Channel 1 when fluorescein was loaded into it (Figure 4A). Then, fluorescein molecules transferred to Channel 2 through the 40% PEG-DA-700 hydrogel barrier (Figure 4B). After 60 min, the green color was clearly observed in Channel 2 as seen with the fluorescence image in Figure 4C. The fluorescence intensity was quantitatively analyzed and plotted in Figure 4D. The fluorescence intensity gradually increased with time from 0 to 60 min in both the 40% PEG-DA-700 hydrogel barrier and Channel 2.

Fick’s second law of diffusion was employed to calculate the diffusivity of fluorescein molecules into the 40% PEG-DA-700 barrier [39]:

(1) $$\frac{\partial C}{\partial t}=D\frac{\partial^{2}C}{\partial x^{2}},$$

where D represents the diffusivity of fluorescein in 40% PEG-DA-700 and C indicates the concentration of fluorescein.

The solution to Equation (1) can be expressed as

(2) $$C\left(x,t\right)=\frac{C_{x}-C_{0}}{C_{s}-C_{0}}=1-erf\left(\frac{x}{2\sqrt{Dt}}\right)=erfc\left(\frac{x}{2\sqrt{Dt}}\right),$$

where $C\left(x=0\right)=C_{s}$ is the concentration of fluorescein at the junction of the porous barrier and the channel containing fluorescein (“Channel 1”), and $C\left(x=\infty \right)=C_{0}=0$ corresponds to the initial concentration of fluorescein in PEG-DA [39]. We assume that $C_{s}$ and $C_{0}$ remain constant over time.

The characteristic diffusion length (*L*) at a given time (*t*) is defined as the distance at which the concentration of the diffusing species reaches 50% of the source concentration ($C_{s}$), and can be approximated as [39]:

(3) L≈(Dt)12.

To determine the diffusivity of fluorescein in the 40% PEG-DA-700 barrier, we acquired time-lapse images of fluorescein diffusion through the hydrogel from 0 min to 10 min using an inverted epifluorescence microscope. The fluorescent time-lapse images were analyzed using ImageJ to obtain the diffusion profiles, which show that the fluorescein molecules diffuse into the porous barrier (Figure 4E). For the duration of the experiment (t = 10 min), since C(x=200 μm)=0, we can assume that there is negligible mass loss from the porous barrier into Channel 2. We plotted the experimentally observed characteristic diffusion length (L) as a function of the square root of time (t12) and performed a linear regression analysis to determine the diffusivity (D). The diffusivity of fluorescein in 40% PEG-DA-700 was estimated to be ~4.6 × 10^−7^ cm^2^/s from the slope (D12) of the fitted curve (L = 6.77951t12, adjusted *R*^2^ = 0.99343) (Figure 4E inset).

## 4. Conclusions

Our simple 3D-printed microfluidic channel with an integrated porous barrier is the first step toward 3D-printed devices capable of more advanced functionalities. We developed a stereolithographic co-printing process to demonstrate the selective diffusion of small ions or fluorescein molecules through a 3D-printed porous barrier. The 3D-printed cross-channel chip and the 3D-printed symmetric-channel chip were successfully printed using PEG-DA-575 and 40% PEG-DA-700, respectively, for the porous barrier and PEG-DA-258 for the microchannels. We were able to confirm the selective hydrogen ion diffusion through the PEG-DA-575 hydrogel barrier by observing the color change of phenol red. Fluorescein diffusion was demonstrated using the 3D-printed symmetric-channel chip, which has a 40% PEG-DA-700 hydrogel barrier in the microchannel. The proposed stereolithographic co-printing process for fabricating selective porous barriers will provide an inexpensive, simple, convenient, and reproducible molecule delivery platform that can be applied in the fields of tissue engineering, drug delivery systems, and biomaterials. 3D-printed hydrogels with larger porosities could potentially be used for filtering particles from bodily fluids such as circulating tumor DNA, exosomes, bacteria, and circulating tumor cells, and in general 3D-printed hydrogels could be used for immobilizing biomolecules within microfluidic devices for biosensing applications. Moreover, the biocompatibility of 3D-printed PEG-DA-258 microdevices as well as PEG-DA-700 structures makes our strategy of printing porous barriers inside a microchannel amenable to the development of cell-based assays and organ-on-chip platforms [40,41].
